# Bilateral Total Ankle Arthroplasty in a Hemophiliac Patient: A Case Report and Literature Review

**DOI:** 10.3390/jcm14124292

**Published:** 2025-06-17

**Authors:** Fernando S. Chiabai de Freitas, Simone Santini, Jose Savio Ferreira Filho, Alexandre L. Godoy Santos, Victor Valderrabano

**Affiliations:** 1 IBRA (International Bone Research Association), Swiss Ortho Center, 4010 Basel, Switzerland; f_chiabai@hotmail.com; 2 Hospital Israelita Albert Einstein, Avenida Albert Einstein 627, Sao Paulo 05652-900, Brazil; alexandrelemegodoy@gmail.com; 3 Department of Orthopaedic and Trauma Surgery, Fondazione Policlinico Campus Bio-Medico, Via Alvaro del Portillo 200, 00128 Rome, Italy; s.santini@unicampus.it; 4 Swiss Ortho Center, Swiss Medical Network, Schmerzklinik Basel, Hirschgässlein 15, 4010 Basel, Switzerland; 5 HEMOSC Avenida Professor Othon Gama D’Eça, 756-Centro, Florianopolis 88015-240, Brazil; saviofer22@gmail.com; 6 Department of Orthopedic Surgery, Faculdade de Medicina, Universidade de São Paulo, Rua Dr Ovídio Pires de Campos 333, Cerqueira Cesar, Sao Paulo 05403-010, Brazil

**Keywords:** hemophilia, ankle osteoarthritis, ankle prosthesis, bilateral ankle arthroplasty, surgical outcomes

## Abstract

**Background:** Ankle osteoarthritis (OA) in hemophiliac patients is an important cause of morbidity. The management of such cases presents unique challenges due to coagulopathy management and surgery technique, especially when it is bilateral. While arthrodesis has traditionally been the procedure of choice for advanced ankle OA, total ankle arthroplasty (TAA) has become an increasingly valuable option due to its potential to restore mobility and function. There is still limited information in the literature regarding the management of these cases, and many questions remain, such as whether to perform surgery in a single stage or two, whether to use anticoagulants or not, and whether to use a tourniquet or not, in addition to the extra precautions required for hemophiliac patients. **Methods:** This work consists of a case report and a narrative literature review. **Results/Case Report**: In this article, we present a surgical case involving a young hemophiliac patient with advanced bilateral ankle osteoarthritis who underwent total ankle arthroplasty in a single procedure. In addition to detailing the surgery, we provide several insights that may assist during the intraoperative and postoperative phases, and we compare our approach with the existing literature. **Conclusions**: With advances in materials and surgical techniques, total ankle arthroplasty has become a viable option, provided that all necessary precautions are meticulously observed. These precautions encompass not only the management of the underlying condition but also careful attention to preoperative, intraoperative, and postoperative care. Further studies with longer follow-up and the use of newer-generation prostheses are still needed.

## 1. Introduction

Osteoarthritis (OA) is a chronic degenerative joint disease that commonly impacts function and the overall quality of life of affected individuals [1]. The ankle is less involved than larger joints such as the knees and hips and usually is secondary to trauma [1,2]. Ankle OA often leads to pain, stiffness, and limited mobility, compromising a patient’s ability to perform daily activities [3]. In patients affected by hemophilia, a genetic bleeding disorder, this condition is caused by the underlying coagulopathy and recurrent joint bleeds, which lead to chronic synovial inflammation, cartilage degradation, and subchondral bone changes [4,5,6].

Hemophiliac arthropathy is a severe complication, frequently culminating in OA in multiple joints, including the ankles [7,8]. Bilateral involvement often results in severe functional limitations, as the loss of ankle mobility and the resulting gait abnormalities can impair the patient’s ability to walk and perform basic activities of daily living. It is estimated that 25–50% of severe hemophilia patients experience bilateral ankle involvement, with the knee, elbow, and ankle being the most commonly affected joints [9].

When non-surgical options are exhausted, surgery should be considered. Arthrodesis has historically been the most commonly used procedure for end-stage ankle osteoarthritis and remains so in many centers. Still, it comes with significant functional limitations, due to loss of joint mobility [10]. This is especially problematic for bilateral cases where the loss of motion severely impairs the patient’s ambulation. However, with advances in surgical techniques and implant materials, total ankle arthroplasty (TAA) has become a highly valued option, making it particularly important in these cases of bilateral involvement. In addition to surgical care, hemophilia patients require special attention due to the increased risk of bleeding, as well as the logistical considerations associated with the procedure. Careful evaluation of various clinical factors is required to optimize outcomes and minimize risks, considering the disorder’s specificities [11,12].

The literature regarding bilateral involvement of the ankle is still lacking, as most studies focus on larger joints, such as the knees, or on single-joint involvement, and the majority have a short follow-up period [13]. Current treatment strategies are often general and not tailored to the unique needs of this population. When planning surgery for a hemophilic patient with bilateral osteoarthritis, several questions arise beyond the choice of procedure. These include the outcomes of each procedure, revision rates, whether to perform the surgery in one or two stages, anticoagulation use, tourniquet application, and the best approach for postoperative care [12,13].

This article provides a literature review on the treatment of bilateral ankle osteoarthritis in hemophilic patients and presents a clinical case of bilateral TAA.

### 1.1. Hemophilia

Hemophilia is an X-linked recessive inherited hemorrhagic disorder. It is caused by a deficiency or dysfunction of coagulation factors VIII and IX, leading to hemophilia A and B, respectively. Studies conducted in large centers, such as those in the United Kingdom, estimate the prevalence of hemophilia A at 12.8 per 100,000 males and hemophilia B at 2.7 per 100,000 males. Epidemiological data on females are limited, as many women are only identified as carriers of the gene after the birth of a son diagnosed with hemophilia [6].

Hemophilia typically starts to manifest in childhood, with recurrent bruising, spontaneous bleeding—particularly into joints, muscles, and tendons—an excessive bleeding following surgical procedures. Joint bleeding (hemarthrosis) is one of the most common and debilitating complications, often resulting in progressive joint damage, reduced mobility, and a marked decline in quality of life [4,5,14].

A thorough family history is essential for diagnosing hemophilia; however, it is important to note that approximately 30% of cases result from spontaneous genetic mutations, with no prior family history of the condition. Other hemorrhagic disorders, such as von Willebrand disease, should be considered in the differential diagnosis of hemophilia. Accurate diagnosis requires appropriate laboratory testing [5,15].

The coagulation profile (coagulogram) serves as an initial screening tool and typically reveals an isolated prolongation of activated partial thromboplastin time (aPTT), with a normal prothrombin time (PT). The definitive diagnosis is established by measuring the activity levels of coagulation factors VIII and IX. By evaluating these factor levels in conjunction with the clinical presentation, hemophilia can be classified as mild, moderate, or severe, as outlined in Table 1 [4,15,16].

Hemophilia treatment has advanced significantly in recent decades, particularly with the development of prophylactic replacement therapy for deficient coagulation factors. This approach has led to considerable improvements in affected individuals’ quality of life and life expectancy [5].

Currently, many studies are exploring gene therapy for hemophilia A or B. New gene therapy approaches, such as lentiviral vectors or gene editing, have shown success in treating other genetic diseases or animal models of hemophilia, but none have been tested in clinical trials for hemophilia. Although promising, these new therapies are not qualified as a cure for the condition, although they may alleviate the treatment burden for patients for several years [17].

### 1.2. Musculoskeletal Manifestations

Osteoarticular changes are common in hemophilia, with osteoarthritis being one of the most significant complications [3]. These changes cause substantial morbidity affecting the patient’s quality of life. The reduced expression of tissue factor in joints and muscles impairs clot formation, thereby promoting recurrent bleeding in these areas [1,18].

Hinge-type joints, such as the knee, ankle, and elbow, are the most commonly affected, whereas multi-axial joints, such as the hip and shoulder, have a lower prevalence of involvement [2]. In severe cases, initial bleeding episodes often occur in early childhood, typically when the child begins to take their first steps [4,19].

Recurrent hemarthroses directly damage cartilage, lead to hemosiderin accumulation, and cause chronic synovitis [8]. The synovial membrane becomes inflamed and thickened, making the joint more susceptible to further bleeding. This initiates a vicious cycle that progressively worsens [5,8].

In chronic cases, radiographic findings may reveal subchondral cysts, sclerosis, joint collapse, loss of articular congruity, and osteophyte formation—all contributing to secondary osteoarthritis. Over the long term, disuse-related changes, such as osteopenia, osteoarthritis, and muscle atrophy, may also occur. It is common to observe on radiographs the presence of arrest lines in the metaphysis, the coarse trabeculation, and a spongy appearance in the epiphysis [18].

Malalignment of the limbs is common, with deformities such as external rotation and valgus deformity of the knee frequently observed. Postural valgus deformities are also common in other joints, including the ankle, elbow, and hip. Varus deformities, on the other hand, are less frequent and typically result from focal bone lesions or premature closure of the growth plates [18].

### 1.3. Preoperative Management

Due to the pain experienced by hemophilic patients resulting from underlying osseous changes and joint degeneration, surgical intervention is often necessary. Prior to surgery, conservative treatment may be attempted, including strengthening exercises, physical therapy, orthotic support, and radiosynovectomy [11]. Once these non-surgical options are exhausted, surgical treatment should be considered.

Arthroscopy for synovectomy, debridement, and osteophyte resection can be performed as a less invasive option prior to more extensive surgical interventions [20]. Ankle arthrodesis remains the most commonly performed procedure in many centers for advanced osteoarthritis; however, with advancements in ankle prosthetics offering improved durability and function, total ankle arthroplasty has become increasingly utilized—even in hemophilic patients [12].

Managing hemophilic patients, particularly in the context of major surgical procedures, requires careful and specialized attention. The medical team involved must work in a coordinated manner, and all components of the treatment plan are rigorously assessed before any intervention. It is strongly recommended that surgeries be performed in specialized centers equipped with experienced, multidisciplinary teams. These should include anesthesiologists, surgeons, nurses, and physiotherapists, working collaboratively to ensure optimal patient care and minimize perioperative risks. Furthermore, a well-equipped laboratory team capable of conducting necessary diagnostic tests is essential to support the overall process [4,5].

The patient should undergo comprehensive serological and infectious disease screening as well as liver and renal function assessments to identify any hemostatic abnormalities and to evaluate the metabolism of medications to be used during the procedure [16,21]. Additionally, preoperative screening for the presence of inhibitors to coagulation factors is essential.

It is crucial to ensure that an adequate supply of coagulation factor concentrates is available for replacement therapy and that the necessary coagulation parameters are maintained at every stage of treatment [5,21].

Several studies recommend preoperative replacement of factors VIII and IX up to 120% immediately before surgery [5,6]. However, the World Federation of Hemophilia (WFH) guidelines recommend maintaining factor levels between 80% and 100% for major surgical procedures [5]. According to the “Brazilian Ministry of Health’s guidelines”, factor replacement should be adjusted to 100% immediately before the induction of anesthesia [16]. Another medication introduced for the management of hemophilic patients is tranexamic acid.

The replacement protocol should follow a strict regimen with continuous monitoring to ensure adequate levels during the postoperative period:

- Maintain factors VIII or IX at 40% to 50% every 8 to 12 h until the third postoperative day;
- Maintain factors VIII or IX at 40% to 50% every 12 h from the fourth to the seventh postoperative day;
- Maintain factors VIII or IX at 40% to 50% every 24 h from the eighth to the fourteenth postoperative day, or until complete removal of sutures [16].

Due to the logistical considerations involved in performing surgery, and the fact that many hemophilic patients have involvement in more than one joint, there is a concern about whether these procedures should be performed in a single surgical session or staged over multiple sessions [22,23,24].

Most studies to date regarding total knee arthroplasty suggest performing bilateral procedures simultaneously. This approach reduces overall costs, particularly those related to coagulation factor replacement. Additionally, it is associated with shorter hospital stays and improved patient rehabilitation outcomes [5,23].

A long-term retrospective study conducted in China found no significant differences in complication rates between cases where multiple joints were treated in a single surgical session and those staged over two procedures [25]. However, the study did not find statistically significant differences in hospitalization duration or improvements in clinical outcomes. It is important to note, however, that this study, which spans over 15 years, did not account for advancements in implant technology or surgical techniques during that period [25].

Antibiotic prophylaxis follows the same principles as in non-hemophilic individuals, with cephalosporins being the most commonly used agents. In cases of cephalosporin allergy, alternatives such as clindamycin or vancomycin may be considered. The most appropriate prophylactic regimen depends on the prevalence of specific pathogens in the region [5].

General anesthesia combined with regional blockade is the most widely accepted approach for anesthetic management. However, in patients with contraindications to this combination, neuroaxial blockade may be used, although these patients require additional precautions and monitoring to ensure safety [5,25].

## 2. Methodology

This work consists of a case report and a narrative literature review.

The patient selected for the study completed and signed the Informed Consent Form (ICF) before undergoing discussion with the Ethics Committee. The study complies with the Declaration of Helsinki.

The literature review was conducted using the PubMed database between November and December 2024, with no date restrictions. The following descriptors were used: hemophilia, ankle osteoarthritis, ankle prosthesis, bilateral ankle arthroplasty, and surgical outcomes.

To select articles, the authors filtered each article by title and abstract, retrieving those that met the criteria of our study. All publications meeting the search criteria were transferred to RefWorks, and all duplicates were removed. The researchers read the titles and abstracts of the initially selected articles and then read the full texts of the articles that passed the final selection process.

**Figure 1:**
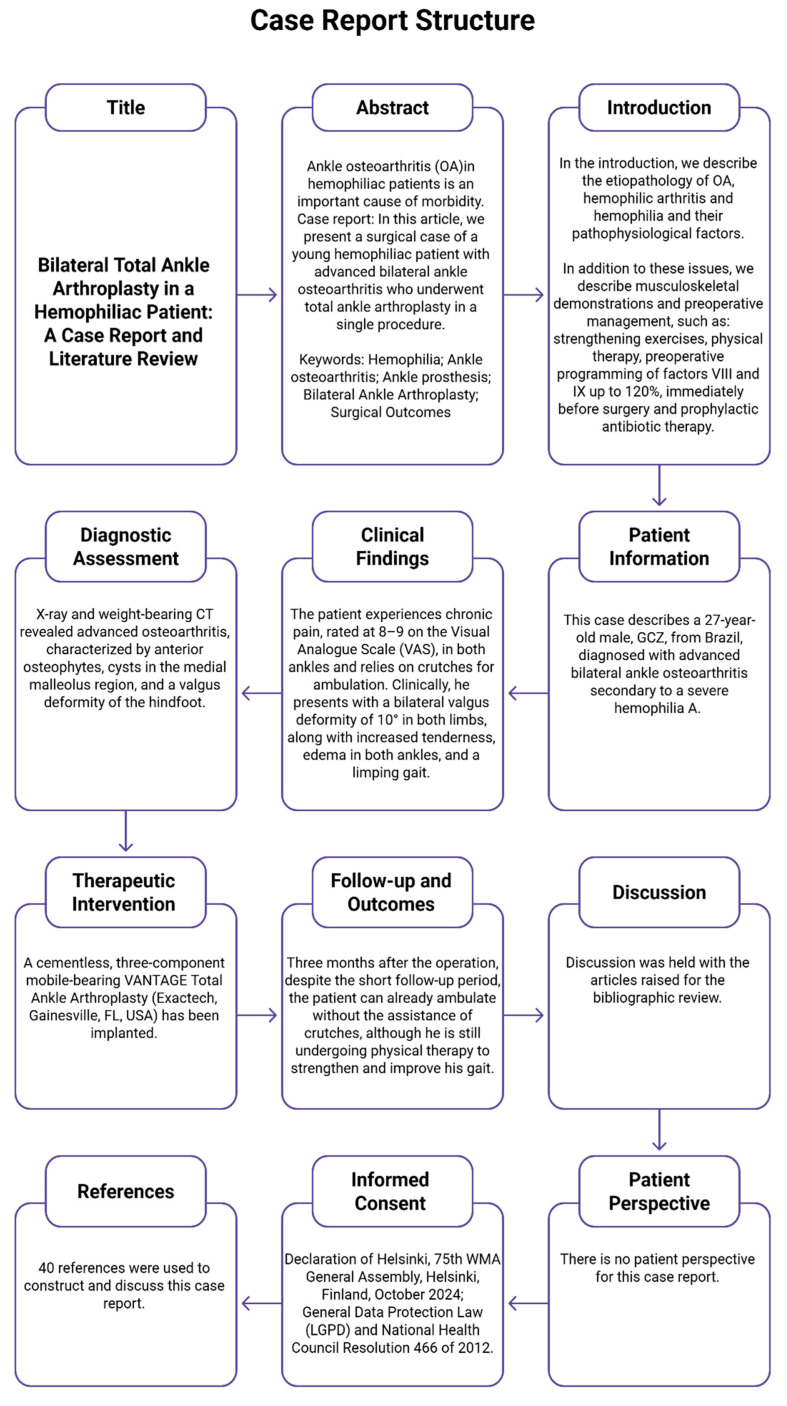
CARE diagram.

For the construction of the case report, the CARE guidelines were followed (Figure 1).

## 3. Case Report

This case describes a 27-year-old Brazilian man, GCZ, diagnosed with advanced bilateral ankle osteoarthritis secondary to severe hemophilia A. He is on a prophylactic factor VIII replacement regimen with factor VIII replacement, receiving 2500 IU three times per week.

**Figure 2:**
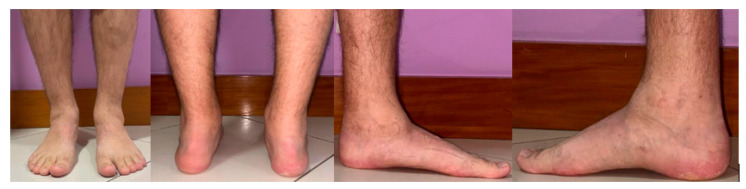
Hemophiliac patient suffering from bilateral ankle osteoarthritis. Preoperative clinical images.

The patient experiences chronic pain, rated 8–9 on the visual analogue scale (VAS), in both ankles and relies on crutches for ambulation. Clinically, he presents with a bilateral 10° valgus deformity, along with increased tenderness, ankle edema, and a limping gait (Figure 2).

His range of motion is significantly limited, with 0° of dorsiflexion and 10° of plantarflexion. Eversion is 20°, and inversion is 10°. The American Orthopaedic Foot and Ankle Society (AOFAS) ankle–hindfoot score is 19/100.

**Figure 3:**
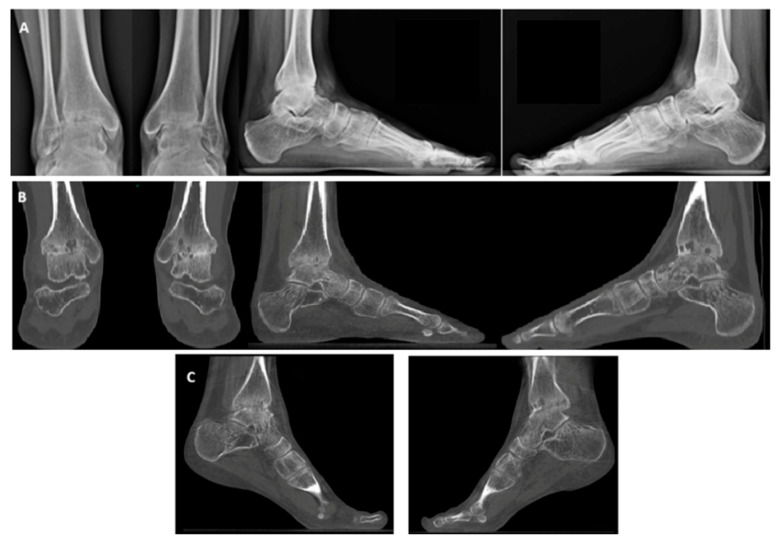
Hemophiliac patient suffering from bilateral ankle osteoarthritis. Preoperative images: X-rays (**A**), weight-bearing CT, AP and side view (**B**), and plantar flexion weight-bearing CT (**C**).

X-ray and weight-bearing CT revealed advanced osteoarthritis, characterized by anterior osteophytes, cysts in the medial malleolus region, and a valgus deformity of the hindfoot (Figure 3). Based on clinical and radiological findings, a decision was made to proceed with bilateral total ankle arthroplasty. Due to logistical considerations and in alignment with most of the literature, a simultaneous bilateral approach was chosen.

The patient received factor VIII replacement therapy according to the protocol, with levels raised to 100% immediately before initiating the procedure. Postoperatively, the factor replacement protocol was maintained as outlined in Table 2 and Table 3.

### 3.1. Surgical Technique

The patient was placed in the dorsal decubitus position under general anesthesia. No tourniquet was applied to facilitate vessel identification and allow for immediate hemostasis. Instead, each vessel was meticulously managed individually to minimize bleeding and reduce the risk of postoperative hemorrhagic complications.

A cementless, three-component, mobile-bearing VANTAGE Total Ankle Arthroplasty (Exactech, Gainesville, FL, USA) was implanted. This fifth-generation system aids in recentering an anteriorly subluxated talus to its anatomical position and reduces shear forces at the bone–prosthesis interface, thereby enhancing implant longevity [26,27,28].

The procedure began with prophylactic percutaneous fixation of the medial malleolus using two cannulated screws. This approach was selected based on imaging studies revealed subchondral cysts in the region, which, together with the osteopenia commonly seen in hemophilic patients, could predispose to fractures. The fixation aimed to prevent intraoperative and postoperative fracture of the medial malleolus.

After fixation of the medial malleolus, an anterior incision was made, and dissection was carried out between the tibialis anterior and the extensor hallucis longus tendon, with careful protection of the neurovascular bundle. Following meticulous dissection and adequate joint exposure, the prosthesis guide was positioned.

**Figure 4:**
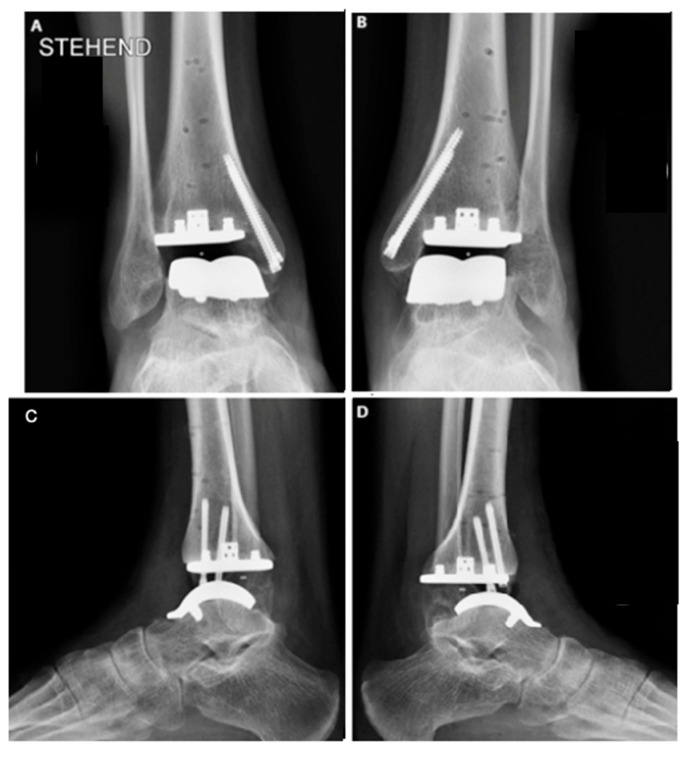
Bilateral total ankle arthroplasty in the hemophiliac patient two weeks after the surgery. VANTAGE Total Ankle Arthroplasty mobile-bearing system with 2 prophylactic screws in medial malleolus in AP and side view, right ankle (**A**,**C**), and left ankle (**B**,**D**).

Once the guides were positioned, fluoroscopy was used to verify the correct alignment of the cuts (Figure 4). Adjustments regarding rotation, varus, valgus, slope, and the amount of bone to be resected were made as necessary. A 5 mm bone resection is typically recommended when using a mobile system; however, in this case, due to the patient’s underlying osteopenia, a more conservative resection was performed.

After the tibial cut, the talar cutting guide was positioned, and the talar cut was performed. Following implantation of the prosthetic components, a suction drain was placed and maintained for 48 h.

### 3.2. Postoperative Management

The patient remained hospitalized for two weeks, during which time coagulation factor levels and inhibitors were closely monitored. No complications occurred during this period and all inhibitor test results were negative. Factor VIII was maintained at appropriate levels in accordance with the protocol established by the hematologist.

Routine pharmacologic thromboprophylaxis is generally not recommended for hemophilic patients; however, it may be considered in cases where the patient has a significantly elevated risk of thromboembolic events, following a thorough individualized risk assessment [5,29]. Therefore, our patient did not undergo thromboprophylaxis with enoxaparin.

According to the literature, early initiation of physiotherapy and appropriate pain management are essential to achieve an optimal range of motion, reduce hematomas and adhesions, and improve pain levels. If no additional procedures are performed alongside the arthroplasty, the patient may begin full weight-bearing immediately, using a walker boot and crutches. However, if osteotomy, arthrodesis, or other procedures are carried out, weight-bearing may need to be restricted to 15 kg for six weeks, with full weight-bearing typically resumed after this period [26,29,30,31].

Postoperative physiotherapy management was conducted according to the literature, beginning on the first postoperative day with early mobilization to improve pain and adhesions, as well as lymphatic drainage to reduce edema. The patient remained non-weight-bearing for 3 days to avoid bruising due to hemophilia; after this period, gait training was initiated at the bedside, barefoot and with full weight-bearing, along with exercises for plantar flexion and dorsiflexion [32].

The patient was also encouraged to perform skiing exercises beside the bed to stretch the gastrocnemius–soleus muscles. Weight-bearing ambulation was then permitted using orthopedic boots and crutches for balance, and this recommendation was maintained after discharge for at least four weeks.

### 3.3. Outcomes

Three months after the operation, despite the short follow-up period, the patient is already able to ambulate without the assistance of crutches, although he is still undergoing physical therapy to strengthen and improve his gait. Improvement can be observed in several aspects, as shown in Table 4 below.

Imaging studies also show that the implant is well positioned, with no detectable loosening or movement, and has good ankle alignment. Further follow-up visits are necessary for long-term evaluation.

## 4. Discussion

Bilateral ankle osteoarthritis is not as rare as it may seem, and its treatment presents a significant challenge, as previously mentioned in this study. Total ankle arthroplasty was performed considering the bilateral involvement and the consequent contraindication of arthrodesis due to severe bilateral functional limitations [10]. A single-stage procedure was chosen to reduce hospitalization and recovery time, as well as to address the logistical requirements for intra- and postoperative management. In addition to the hematological changes, it is important to consider the osseous alterations that these patients experience, such as malalignments and bone weakness, which can lead to complications if not properly addressed. In this case, less bone was removed during tibial osteotomy, as we anticipated some degree of bone accommodation and potential loosening of the implant could occur. Furthermore, due to the reported bone weakness and the presence of cysts observed in preoperative imaging, the decision was made to place screws in the medial malleolus to prevent fractures in the region. Before the procedure begins, it is important to make sure that everything is ready to go to minimize intraoperative complications, time of surgery, and further complications.

The patient demonstrated significant improvement in range of motion, function, and pain levels. However, further pain relief is expected, as the patient is only in the third month following the operation, corresponding to the classic three-month postoperative adaptation phase in foot and ankle surgery, as described by Santini and colleagues. The patient is still undergoing physical therapy to improve strength and gait adaptation [33]. No complications have been observed so far.

A retrospective French study analyzed 29 cases from 2006 to 2019. Short-term complications were observed in 11 of the cases. Six of these involved hematomas that occurred within the first 5 days following the operation, four of which were attributed to the use of prophylactic heparin or ketoprofen due to prescription errors. Additionally, two patients experienced medial malleolar fractures, one occurring intraoperatively and the other on the fourth postoperative day. In the long-term follow-up, five cases required revision surgery. Four of these involved the use of a prosthesis that had been withdrawn from the market due to poor outcomes. However, significant improvements were noted in both AOFAS scores and in patients’ overall mobility [29].

Another study conducted in Zurich, with a long-term follow-up of 10 years, evaluated 17 total ankle arthroplasties performed between 1998 and 2012. The five-year survival rate was 97%, with 85% at 10 years and 70% at 15 years, which is comparable to outcomes observed in studies involving patients without hemophilia or with inflammatory arthropathies [13,30,34].

A systematic review with meta-analysis assessed 21 studies on ankle arthrodesis and prosthesis in hemophilic patients, aiming to compare the outcomes of these interventions. The findings revealed similar complication rates between the two groups. In the TAA group, the most common complications were aseptic loosening (5.8%) and wound-related issues (5.4%). In the AA group, the most common complications were wound-related issues (9.8%) and non-union (7.9%). When evaluating the AOFAS score and the VAS score, the results were also comparable, demonstrating significant improvement in both groups. Nevertheless, this study was limited by a small sample size and the predominance of retrospective studies, which were highly heterogeneous and of low evidence quality [35].

Mussawy et al. [36] also evaluated the outcomes by comparing TAA with AA in a cohort study, where they compared 9 patients (11 ankles) who underwent arthrodesis with 10 patients (11 ankles) who received a prosthesis. The results showed significant improvement in satisfaction levels and pain relief, with similar outcomes between the groups. However, patients who underwent arthroplasty had a higher rate of deep infection, even when compared to existing studies in the literature. In contrast, patients undergoing arthrodesis experienced more frequent complications, with non-union being the most prominent.

Valderrabano et al. [28] conducted a study on 36 ankles of patients with coagulation disorders who underwent ankle arthroplasty between 2002 and 2013, with an average follow-up of 6.3 years. These patients were compared to a control group consisting of 72 individuals with post-traumatic osteoarthritis and 33 with rheumatoid osteoarthritis of the ankle. The study showed a significant improvement in pain and mobility scores compared to the control group, with low rates of postoperative complications in the medium term, requiring only three revision surgeries. Postoperative radiographic evaluations confirmed proper alignment of the prostheses in all patients [37].

In the assessment of mid-term outcomes with an average follow-up period of 4.4 years, Ascensio evaluated 32 total ankle arthroplasties (TAA) in 21 patients who underwent surgery between 2002 and 2009. The study analyzed clinical outcomes using the AOFAS and AFCP scores, as well as radiographic results. Regarding clinical outcomes, the AOFAS score improved from 40.2 to 83.4, showing progression similar to that observed in previously cited studies. Radiographically, Ascensio’s results were consistent with those of Valderrabano, with all prostheses remaining stable and properly positioned in every case at the last examination, with significant improvement in range of motion [38,39].

Most of the studies with longer follow-up periods have focused on third-generation prostheses, such as Hintegra, STAR, Mobility, and InBone. However, more advanced implants, including fourth- and even fifth-generation models like VANTAGE, are now available. These newer prostheses are expected to yield even better results in long-term studies once data become available [13,39,40].

The fact that this study is a case report with only a single case and a short follow-up time can be considered a limitation of the study. Also, there are few studies in the literature on this topic due to the disease being uncommon and the need for multidisciplinary monitoring with an experienced surgeon.

## 5. Conclusions

With advancements in surgical materials and techniques, total ankle arthroplasty has become a feasible option, provided that all necessary precautions are meticulously followed. These precautions encompass not only the management of the underlying condition but also careful attention to preoperative, intraoperative, and postoperative care. It is important to emphasize that this is a challenging procedure, which should be performed exclusively by highly experienced foot and ankle surgeons. Furthermore, additional studies with longer follow-up, utilizing prostheses from newer generations, are still needed. A key message for surgeons performing this procedure is as follows: Do not initiate the procedure before ensuring that all aspects have been thoroughly assessed.

## Figures and Tables

**Table 1:** Relationship between bleeding severity and clotting factor level.

Severity	Clotting Factor Level	Bleeding Episodes
Severe	<1 IU dL^−1^ (<0.01 IU mL^−1^) or <1% of normal	Spontaneous bleeding into joints or muscles, occurring predominantly in the absence of any identifiable hemostatic challenge.
Moderate	1–5 IU dL^−1^ (0.01–0.05 IU mL^−1^) or 1–5% of normal	Occasional spontaneous bleeding: prolonged bleeding with minor trauma or surgery.
Mild	5–40 IU dL^−1^ (0.05–0.40 IU mL^−1^) or 5 to <40% of normal	Severe bleeding with major trauma or surgery. Spontaneous bleeding is rare.

Source: Berntorp et al., 2021 [4].

**Table 2:** Factor VIII protocol infusion.

Time of Infusion	Dose	Reason
Surgery	3500	Infusion 00:30 h before surgery
	2000	Infusion 08:00 h after surgery
D1 to D7	2000	Infusion every 12:00 h after surgery
D7 to D14	2000	Infusions every 24 h until 14 days after surgery or until removal of the suture
D14 + 2	2000	Infusion after 14 days, until stitches removed
Prophylaxis	2500	Primary prophylaxis 3 times a week

**Table 3:** Tranexamic acid protocol infusion.

Time of Infusion	Dose	Reason
D1 to D5	1000 mg	Every 8 h

**Table 4:** Postoperative changes in 3 months.

Evaluation	Preoperative	Postoperative
VAS	8–9 points	3–4 points
AOFAS	21 points	71 points
ROM dorsiflexion	0°	10°
ROM plantar flexion	10°	25°
Angulation	10° valgus	Neutral

Legend: VAS—visual analogue scale; AOFAS—American Orthopaedic Foot and Ankle Society; ROM—range of motion.

## Data Availability

The original contributions presented in the study are included in the article, further inquiries can be directed to the corresponding authors.
